# Group prenatal care experiences among pregnant women in a Bangladeshi community

**DOI:** 10.1371/journal.pone.0218169

**Published:** 2019-06-12

**Authors:** Marufa Sultana, Nausad Ali, Raisul Akram, Tania Jahir, Rashidul Alam Mahumud, Abdur Razzaque Sarker, Ziaul Islam

**Affiliations:** 1 Nutrition and Clinical Services Division, International Centre for Diarrheal Disease Research, Bangladesh (icddr,b), Dhaka, Bangladesh; 2 School of Health and Social Development, Deakin University, Melbourne, Australia; 3 Health System and Population Studies Division, International Centre for Diarrheal Disease Research, Bangladesh (icddr,b), Dhaka, Bangladesh; 4 Infectious Disease Division, International Centre for Diarrheal Disease Research, Bangladesh (icddr,b), Dhaka, Bangladesh; 5 School of Commerce, University of Southern Queensland, Toowoomba, Australia; 6 Department of Management Science, University of Strathclyde, Glasgow, United Kingdom; Kwame Nkrumah University of Science and Technology, GHANA

## Abstract

**Background:**

Complications during pregnancy, childbirth, and following delivery remain significant challenges that contribute to maternal morbidity and mortality, thus affecting health systems worldwide. Group prenatal care (GPC) is an integrated approach incorporating peer support and health education that provides prenatal care in a group setting. The GPC approach was piloted in a district of Bangladesh to measure the feasibility and effectiveness of GPC compared to individual care. Understanding the experiences of women of receiving this grouped care approach is crucial to understand the perspectives, perception, and acceptability of the programme among mothers, which are lack in Bangladesh. The objective of the present study was to understand the core experiences and perspectives of mothers who participated in GPC sessions during their pregnancy period.

**Methods:**

A qualitative research approach was used to understand the experiences of women receiving GPC. A total of 21 in-depth interviews were conducted in this study targeting pregnant mothers who attended all recommended GPC sessions. Face-to-face interviews were conducted by trained and experienced interviewers using a specific interview guideline to achieve detailed responses. Thematic analysis was conducted to analyse the data.

**Results:**

Mothers appreciated receiving pregnancy care in group setting and expressed their preferences towards GPC compared to individual care. Themes included the comprehensiveness of GPC, prescheduled appointments and reduced waiting time, social gathering, coping with common discomforts, relationship with service providers, birth preparedness, and recommendations from participating mothers. The themes conveyed overall positive experiences of the participating mothers, with suggestions for further betterment of the programme. Nevertheless, the reported experiences of women involved in the study suggests that the inclusion of a specialist in group care, post-partum care, and family planning advice will be more beneficial in the GPC model.

**Conclusions:**

The overall experiences of the women in the present study suggest that GPC is helpful for them, and it is useful to reduce complications during pregnancy. The GPC model promises movement towards family-supported care, as explained by the participants.

## Background

Complications during pregnancy, childbirth, and following delivery remain significant challenges that contribute to maternal mortality and affect health systems worldwide [1]. Maternal health care has been considered one of the key elements of the Millennium Development Goals (MDGs) and the more recent Sustainable Development Goals (SDGs), though progress on improving maternal health indicators remains slower than expected in many developing countries [2]. According to the Bangladesh Maternal Mortality and Health Care Survey (BMMS), the estimated maternal mortality ratio (MMR) is 196/100,000 live births in 2016, which accounts for around 13% of total adult female deaths and has shown no significant change since 2010 [3]. Notably, the majority of maternal and neonatal deaths were concentrated among the poor and in rural areas, and largely occurred due to lack of service availability, accessibility, affordability, or poor quality of services [2,4].

Bangladesh is recognised as a role model for other developing countries in achieving the MDGs. Despite this, the maternal and neonatal mortality rate remains high, and nearly 62% of deliveries occur at home, which leads to an increased risk of maternal and neonatal morbidity and mortality [5]. Notably, maternal deaths are primarily caused by postpartum haemorrhage (31%), eclampsia (20%), and abortion (15%) [6,7]. A recent demographic and health survey indicated that the percentage of mothers receiving the recommended number of antenatal care (ANC) and postnatal care (PNC) visits (4 or more) from medically trained providers is very low (31% and 35%, respectively), while only 37% of deliveries are performed in a health facility [5]. Although available data indicate signs of improvement compared to preceding years, such improvements indicate the existence of inequality [2,5]. Additional efforts are inevitable to ensure improved quality, coverage, and content of maternal care so that all strata of society receive similar benefits in terms of maternal health services.

The quality of maternal care during pregnancy, at the time of delivery, and in the post-delivery period are important for the survival and well-being of both mothers and newborns [7]. Prenatal care is a form of preventive care that provides regular check-ups, treatment, and advice to prevent potential health problems throughout pregnancy, which is beneficial for both mothers and their children [2,8]. Traditionally, pregnant mothers receive prenatal care individually from providers in private examination rooms in public, private, or non-governmental organisation (NGOs) facilities in Bangladesh [7]. Previous studies found that traditional individual prenatal care can be supplemented by group prenatal care (GPC), which facilitates support networks, social interaction, and additional education, while having a positive impact on perinatal outcomes [9–12]. GPC is an integrated approach that provides prenatal care in a group setting by incorporating peer support and health education, which encourages free exchange and develops mutual support among peers [13]. However, such group meetings with peers of the same gestational age are less common in Bangladesh [7].

As a consequence of GPC’s success in improving maternal health care utilisation and outcomes in other settings, a project of “introduction of GPC in Bangladesh” was piloted to measure the feasibility and effectiveness of GPC instead of individual care. In this project, pregnant women put into groups and attended sessions following regular physical assessments on scheduled ANC visits as an intervention. Discussion topics mainly included healthy eating, pregnancy concerns, self-care, substance abuse, childbirth preparation, breastfeeding, contraception, and parenting strategies.

Several studies explored women’s experience of GPC in different settings. For instance, Baldwin; and Ickovics et al. reported quantitative scores of knowledge and satisfaction among women that participated in grouped care [14,15]. Moreover, two studies in an African setting reported qualitative findings on the GPC experiences of mothers through focus group discussions [16] and in-depth interviews [12]; and reported positive experiences among mothers. Existing studies have been conducted in either developed regions or even resource-poor settings; nonetheless, such studies are lack for the South Asian region, which includes Bangladesh. Understanding the experiences of women receiving group care is therefore crucial to understanding the perspectives, perception, and acceptability of the programme among mothers. The findings of the present study will help to inform policy regarding the experiences, expectations, and perceptions of mothers regarding what should be added, removed, or modified from the GPC programme. The present study also considers the social and cultural aspects of including GPC in the existing health care system. As such, the objective of the present study is to understand the core experience of the women that participated in GPC sessions.

## Materials and methods

A qualitative research approach was used to understand the experiences of women using GPC. This approach provides more in-depth and circumstantial evidence [17] while providing a comprehensive summary of events in the usual language of the participants [18]. As such, it enables us to explore the experience gained by pregnant women through participation in GPC sessions with other pregnant women, and to understand motivations for the utilisation of prenatal with postnatal care among them.

### Study setting

This qualitative study is nested within a project that implemented GPC in Bangladesh. The project was conducted in a government-run Maternal and Child Welfare Centre (MCWC) situated in the municipal area of Chandpur District. The municipality has a population of 95,000, of which approximately 50% are female. Among the total number of pregnant women listed in this municipality, approximately 7% received at least four ANC sessions from qualified health care providers. Recruitment was conducted in this single centre to compare both intervention and control groups, as it serves a population with similar socio-demographic characteristics.

### Study population

Pregnant women within 20 months of gestational age who sought prenatal care in the study site were recruited in the study as per the study protocol [7]. Women assigned to the GPC were considered the “intervention group”, while those that received the standard existing care were considered the “control group”. A total of 697 pregnant women with specific inclusion criterion were recruited and randomised for placement into either the intervention or control group. A total of 300 pregnant women participated in at least one GPC session. Each group consisted of 6–10 pregnant women who met together 4–6 times throughout their pregnancy period for general physical assessments and attending group sessions [7]. A total of 125 sessions were organised throughout the intervention period of the project. The sessions were conducted in a separate room involving the standard prenatal assessment followed by group discussions. After physical assessments were performed, GPC began with an educational discussion session for approximately 30–40 minutes, where health education related to the prenatal period, delivery, and postnatal care was mainly emphasised. The sessions were initially facilitated by study nurses, while experienced mothers later discussed their health issues, thus ensuring peer education. The control group received the current standard pregnancy care. The details of this intervention procedure have been described elsewhere [7].

### Sampling procedure and sample size

Among the GPC participants, the study aimed to include those who attended at least four GPC sessions along with other characteristics (age, birth outcome, and education). The rationale of this selection process was to identify women who could best provide rich and in-depth information on their experiences with the services provided through GPC. As such, a total of 107 pregnant women who joined at least four sessions were purposively selected; among them, 21 were selected for the in-depth interviews. The final sample size was determined by informational deliberation i.e. data saturation while no new information was generated by the respondents [17,19]. As a result, a total of 21 interviews were conducted for the present study.

### Data collection

Considering the convenience and availability of the participants, we decided to conduct in-depth interviews. Potential study participants were first contacted via telephone and the purpose of the study was then briefly described to them. An interview date was fixed for the candidates who agreed to participate in the in-depth interview. The interviewers deployed to conduct the in-depth interviews (IDIs) were well trained on the guidelines and had significant experience in using qualitative data collection tools. Data were collected from February to April 2016. The time of the data collection was arranged considering the convenience of the participants, and each interview lasted for approximately 45–60 minutes. Interviews were conducted in Bangla, and each of the discussions between the participant and interviewer was recorded using an audio recorder with the permission of the respondents. Data were collected using a specific interview guideline. This interview guideline was adopted from published qualitative literature based on GPC and then finalised by the researchers involved in the study, considering both cultural and socio-demographic aspect. Both written and verbal informed consent were obtained from all participants prior to the data collection. Participants were first asked to provide written informed consent to participate, and then verbal consent was recorded at the beginning of the interviews. Questions were initially broad, according to the guidelines, and later probing was applied to achieve more detailed responses.

### Data analysis

Thematic analysis was conducted to ensure the methodological accuracy and transparency of the analysis of qualitative data [20]. To perform the thematic analysis, several phases were followed according to the methodology adopted by Braun and Clarke [21], which included becoming familiar with the data, generating initial codes, defining and reviewing themes, and drawing overall interpretations.

Transcriptions were made from each of the recorded interviews by the interviewers in native language (Bangla), and the reading of transcripts with field notes was performed for an overall understanding. All transcripts were then translated into English by a bilingual translator who was directly involved in the study. All transcriptions and translations were checked twice by the other investigators. Data were manually coded according to meaningful statements (issues, highlights, concerns, and accomplishments) in relation to GPC experience, and then categorised by team investigators. Contents were compared across codes, and key concepts were recognised from where core themes were identified. The lead and senior authors cross-checked the themes for common agreement and refined the identified themes. The investigators finally listed some specific themes based on the guidelines and code categories that included participants’ own perceptions, personal beliefs, and understanding. Throughout the intervention period, investigators visited several times, observed the session’s activities and took notes. While generating themes, these notes were also considered. Finally, an evaluation of the themes with a re-reading of the interviews was performed to ensure that the insistence, meaning, and perception of the participants were precisely captured.

### Ethical approval

The protocol of the present study was approved by the Research Review Committee (RRC) and the Ethical Review Committee (ERC) of icddr,b (PR-14119). All study participants provided both written and verbal consent prior to the interview. Detailed study objectives and the contents of consent forms for voluntary participation were explained to each participant prior obtaining their consent.

## Results

A total of 21 interviews were conducted up to data saturation with no refusal. Table 1 presents the basic demographic characteristics of the participants. The majority of interviewed women were aged between <20 and 24 years (62%), and eight women (38%) were experiencing their first pregnancy. All respondents had formal education, while most completed up to the secondary level education (n = 13).

**Table 1:** Background characteristics of mothers who participated in the interviews.

Characteristics	Frequency (n)	Percentage (%)
**Age in years**		
*Less than 20*	2	09.52
*20–24*	13	61.90
*More than 24*	6	28.57
**Mother for the first time**	8	38.10
**Education level**		
*Up to primary*	2	09.52
*Secondary*	13	61.90
*Higher secondary*	5	23.81
*Higher*	1	04.76
**Monthly household income (BDT)**		
*Less than 15*,*000*	7	33.33
*15*,*000–20*,*000*	5	23.81
*20*,*001–30*,*000*	5	23.81
*More than 30000*	4	19.05

All elucidations provided by the participants were classified into seven different themes that described the perceptions and experiences of women who participated in the GPC sessions. These themes are presented in the following sections using the participants’ direct voice to explore the in-depth context and detailed meaning of specific themes that optimally described their experiences. Theme description is anonymised and quoted in italics.

### Theme 1: Comprehensiveness of pregnancy care services

GPC service participants were abounded with all of the required information and services they needed during their pregnancy. They were able to learn common things during pregnancy in different and meaningful ways, which make them more conscious about their reproductive health. These points were reflected through the following sub-themes.

### “Getting all of the necessary services at the right time”

The majority of participants mentioned that they received nearly all of the required services simultaneously from GPC. Most participants reported that, along with the regular physical assessment, detailed explanation and discussion on various topics (i.e., general discomforts, complications and danger signs during pregnancy, dietary suggestions, regular physical activities, safe daily activities, and newborn child care) were delivered in the session, which had made the discussion session livelier and self-contained. The content of each session was designed so that each group consisted of mothers of the same gestational age, thus enabling them to learn about all possible issues according to their respective gestational period. To describe the services and contents of the GPC sessions, a 20-year-old mother stated that, “*In GPC*, *we talked with nurse*. *They did physical check-ups*, *measured blood pressure*, *and observed the movement and position of the baby and sent us to doctor’s room in case of any difficulties*, *and the doctor prescribed medicine(s) if needed*.” She also emphasised the support that she received from GPC even after childbirth. She said; “*One of the good things about GPC is that they followed up the newborn’s condition for two months and took information on the weight of the baby*, *advised about vaccination time and its importance*, *and so on*. *If I did not participate in the GPC sessions*, *then I would not know about these important things that need to be taken care*. *But now*, *GPC has made me free from these anxieties*.”

Another mother who attended all of the recommended GPC sessions said that, “*We have come to know almost all pregnancy-related health issues from GPC*. *They informed us on how to maintain a nutritious diet*, *as well as what to do and what not to do—all of the things we needed to know*. *They also said to make a phone call (mobile) to them if we face any problem(s)*.”

### “Learning things in different ways”

Pregnant mothers perceived that they had learnt topics in a different way from the general antenatal care visits. They participated in discussions with nurses and could also learn many topics visually, as the moderators used video footage and pictures to make them better understand the danger signs of pregnancy and other relevant health issues.

One of the mothers (aged 22 years) explained the different ways of learning through the following statement, “*One more thing is that the moderator also showed many things through videos*. *What to do*, *what to eat*, *what are the danger signs in pregnancy*, *and where to go for safe delivery; all of these were shown in pictures*. *In the session*, *all of these were discussed along with the pictures*.”

As pregnant women sat together during GPC sessions, they could learn many things by discussing topics with peers who had a previous history of delivery. A first-time mother (aged 21) stated; “*In these sessions*, *I learnt so many things from other experienced mothers*, *such as the possibility of excessive bleeding*, *convulsion*, *and other issues that could occur during the time of pregnancy*.”

Few mothers reported that they acquired all of the required information and received services at the exact time they were required.

### Theme 2: Prescheduled appointments, reminders and reduction of waiting time

Prescheduled appointments and mobile communication reminders helped mothers to participate in each GPC session with scheduled ANC visits. Minimal waiting time reduced additional suffering for participating mothers.

### “Reduced waiting time and minimised suffering”

“*I may have had to wait for a long time or had to return back home without a check-up*. *Right after reaching at the facility*, *the nurse allowed us to sit in a room*, *received our cards [recruited as participant]*, *and made arrangements for a general physical assessment by the doctor*. *This was a benefit of this programme*. *In a word*, *I never had to return without a check-up*”. This statement, made by a 24-year-old mother, expanded the importance of group care to reduced waiting time. In contrast to standard care, participants reported minimal waiting time as they had definite appointments in advance. GPC assigned participants to a particular group with the specific time slots for the session, which allowed them to receive regular check-ups with minimal waiting times, thus satisfying the pregnant women.

One-third of the participants noticed some differences in regular check-ups in GPC compared to those of standard care. One mother, aged 29 years, stated that, “*There are surely some differences from other ways/places for physical check-ups*. *We have to wait in a long queue to visit a doctor in other places*, *but through GPC*, *we gathered in a room and discussed many things*.”

Another first-time mother also emphasised this theme: “*Furthermore*, *after submitting our cards [GPC group]*, *we participated in the discussion in a room and learnt many things while other mothers were waiting in the queue for the check-up*.”

### “Almost impossible to forget the check-up date”

The majority of women in GPC overwhelmingly liked two things most; one was sitting with other mothers in the same place, while the other was getting reminder calls via telephone prior to the check-up date. They were delighted by this and felt the importance of antenatal care by receiving phone calls to remind them about their scheduled check-up date. One respondent, aged 20 years, explained her opinion by saying, “*Every month I received two phone calls from them to remind me of the check-up dates; the first call was 10 or 12 days before and the other call was 1 day prior to my check-up date*. *Viewing the number on my phone screen*, *I could remember my scheduled check-up date*. *I was careful about this*.” Another mother (aged 30 years) stated, “*Before our check-up date*, *we got phone calls from the team*. *I went for a check-up when they called me*. *I might have forgotten my date if they had not called me*”

### Theme 3: Social gathering to reduce stresses and solitudes

Participating mothers believed that sitting together and participating in group discussions helped to relieve them of stress and tension. They also reported that GPC allowed them to obtain answers to many unresolved questions. All of these points emerge in the following sub-themes.

### “We sit together; we learn together”

Over time, mothers felt comfortable in the group as relationships grew up among peers, discussion topics evolved, and they learnt by discussing with each other. Moreover, being a member of a group reduced their anxiety and feeling of being alone during the pregnancy period. They received friendly assistance, even from peers, to direct them to the doctor’s room. One 20-year-old mother stated, “*It was beneficial to come across all the mothers together*. *I might not have understood many things or felt lonely*, *or I might not have reached the office on time or been able to find the doctor*. *Now*, *through GPC*, *they helped us find the doctor*”.

A few mothers reported that they enjoyed sharing their pregnancy experiences with others who had similar experiences and concerns. Emphasising on that topic, one 30-year-old mother, “*I benefitted by getting in touch with all the mothers*. *Other pregnant women in my group asked different questions*. *Those topics were unknown to me and I learnt by hearing the solutions*”

### “Getting answers to questions without asking them”

Women’s active participation in discussions and their GPC experience varied widely. Some participants rarely spoke during sessions; however, they were attentive to the discussions and acquired knowledge. One mother stated, “*In the group discussion when I talked about a problem of mine*, *some of the others knew about it and experienced the same*. *Similarly*, *I also learnt about many things by hearing about problems faced by other peers*” (mother, aged 24 years).

Another mother, aged 33 years, also stated that, “*It was helpful to have all the mothers in one place at one time*. *We could know about any issue by hearing from others*. *Similarly*, *other mothers could also learn something by hearing from us*.”

The opportunity to learn from other members through group discussions was a noticeable benefit of GPC. The pregnant women believed that they benefitted from the experienced mothers in their group. A first-time mother (19 years old) said; “*Getting together in GPC*, *we learnt many things from others*. *I was experiencing first-time pregnancy and there were other pregnant women who experienced it four or more times*. *They discussed and shared their experiences and we learnt many things from them*.” She also believes that by attending the group discussions, she received many answers for questions she did not even ask; “*Sometimes*, *in the group sessions*, *there was a time limitation to ask individual questions*. *By asking a common question for any mother*, *we all got the answer*.”

One session participant (aged 20 years) believed that every single moment she spent on GPC was effective, even though the moderator was not present after the completion of the session. In her words, “*When the moderator was not present*, *mothers continued to discuss various informative matters*, *and we learnt from them*.”

From GPC sessions, mothers learnt many things about their regular physical activities, food intake, and recommendations for taking rest. Mothers commented that they were more alert about eating well and exercising because of GPC. One mother (aged 30) stated that the group sessions helped her to be healthier through maintaining the recommended behaviours. In her voice: “*In the meeting*, *lots of things were discussed*, *like what to eat*, *not to lift heavy weight*, *consuming sufficient food and water*, *and so on*”

### Theme 4: Coping with common discomforts during pregnancy

It was difficult for many of the mothers—particularly those experiencing first-time pregnancy—to differentiate pregnancy-related problems and whether to consider or ignore them due to lack of proper knowledge. In GPC sessions, all of these problems were discussed to make mothers aware of common discomforts and danger signs.

### “Received effective information on common pregnancy-related discomfort”

Mothers reported that the opportunity to connect and share their pregnancy experiences, common discomforts, and other general knowledge about pregnancy were a very important part of GPC. They believe that this information would help them in the future as well. A first-time mother, aged 24, explained, “*In the sessions*, *the information they provided on pregnancy care was so effective that I believe I will not face any problems in the future*.”

Another mother (aged 35 years) said, “*If they spread the information that was taught in the GPC to all pregnant mothers rather than just the GPC sessions*, *then it will be very beneficial for all*.”

### “Conscious about symptoms: Whether to ignore or consider”

Sometimes, due to a lack of awareness regarding the common symptoms of pregnancy, many mothers suffered greatly, especially those who were experiencing first-time pregnancy. To reduce this suffering, GPC clearly taught pregnant women about common discomfort, complications, and danger signs requiring medical treatment. One 20-year-old mother explained, “*Some of the problems*, *previously*, *I ignored as minor difficulties and considered that no treatment will be required and that I will be fine anyway; such as if I had problems with water retention or leg swelling*. *Now*, *after attending GPC sessions*, *I came to know that this is not a negligible issue as it may head towards danger signs*.”

Later in the interview, she also acknowledged; “*Conversely*, *we sometimes go to consult the doctor with some common problems*, *like intermittent abdominal pain*. *But in the session*, *we learnt that it is not a problem and rather a usual occurrence during pregnancy*.”

### Theme 5: Upturns of provider-patient and family relationships through GPC

Due to an insufficient number of health professionals with high workload, doctors may not be able to devote a sufficient amount of time to patients in some cases. Moreover, reproductive health remains a taboo in some people, who may feel shy to explain their problems to doctors and family members. To some extent, GPC mitigated these barriers, which were mentioned by the mothers.

### “Feeling free to discuss with health workers and other peers”

Women became knowledgeable, developed greater awareness regarding their own health, and learnt how to care themselves and their upcoming newborns. The social connections and support from providers were identified as beneficial to the women. While discussing the services and attitude of the service providers, a mother (aged 20 years) said, “*People from the GPC treated us great*. *They all talked in a very polite manner*. *Actually*, *patients’ suffering from illness goes away if doctors behave well with them*. *All the people of the GPC were well-mannered*.”

Another first-time mother (aged 24 years) added to this point by saying, “*They discussed many health issues about pregnancy*. *I asked about my problems and they tried to give me solutions*. *Basically*, *they talked about the solutions in more detail*, *which helped me to understand clearly*.”

### “Getting supports from family members”

Though husbands and other guardians such as parents and in-laws were not included in the GPC discussions, mothers reported that they shared the GPC session discussions with their husbands, which changed their husbands’ perceptions about prenatal care. They also reported that they received extra care from their partners since they became more conscious about their pregnancy health issues. One mother (aged 30 years) said, “*Participation in the GPC sessions was beneficial*, *as we did not get such services before*. *During the time of my pregnancy*, *my husband took care of me after hearing about the various aspects of the GPC session discussions from me*.”

Few participants reported that their mothers-in-law, who were previously reluctant about regular check-ups, became more conscious about pregnancy care after observing the GPC procedure. According to the statement of a first-time mother, “*It was good that my mother-in-law—who did not allow me to go for check-ups earlier—became motivated after taking her to the facility*, *and she did not restrict me from going for further check-ups*. *This is how I benefitted*.”

### Theme 6: Birth preparedness and confidence to tackle situations

GPC sessions also emphasised birth preparedness for timely access to skilled delivery care services. In GPC sessions, mothers were taught to be psychologically and financially prepared, while arranging for suitable vehicles to reach to the nearest delivery centres. Participants’ perceptions on this issue are collated in this theme.

### “Feeling prepared for labour and delivery”

Participants reported that they took preparation prior to delivery, which made them confident about their labour and the postpartum period. They mentioned that this happened as a result of participating in GPC sessions, as they would not have otherwise considered birth preparedness. They also believed that their partners also felt the same, as a 29 years’ mother stated: “*In GPC sessions*, *various danger signs were discussed with pictures*, *and suggestions were provided on when to consult a doctor*. *They were also advised to deliver with the presence of a skilled birth attendant or nurse*, *and suggested that someone with a matching blood group be present prior to delivery*, *as blood transfusion may be required during/after delivery*.”

### “Arranging vehicles and money for delivery care”

Participants were suggested to arrange suitable mode of transportation and sufficient money on hand for the delivery. To mention this topic a mother (aged 30 years) commented, “*They advised us to visit health centre in case of excessive bleeding or coming hands or legs instead of head during delivery*. *For managing this situation*, *early preparation*, *arranging transportation and depositing money is very important those also were taught in the sessions*.”

Moreover, to explain the effectiveness of the information that they got received the GPC, one mother (aged 20 years) stated that, “*We learnt a lots of things from the GPC session*, *such as a minimum of four check-ups*, *the importance of blood grouping*, *saving some money for delivery purposes*, *and managing transportation in case of emergency*. *If the delivery centre is far away*, *confirming transportation is very much important*.”

### Theme 7: Recommendations for prospective mothers

There were also some recommendations made by participants regarding the improvement of GPC services. Some points were elicited by them that require improvement. Broader discussions about family planning methods, including diagnostic and delivery services, were recommended by GPC participants. All of these recommendations are explained under the sub-themes.

### “GPC is helpful for pregnant women and would be more beneficial with some moderation”

Overall, the women were satisfied with their GPC experience, which is reflected through this statement made by a first-time mother (aged 20 years), “*I wish GPC would always continue so that many mothers would benefit like me*.”

When they were asked about any aspects of the model they would like to change, they had made a few suggestions for improvement of the content and process. One mother (aged 24) suggested: “*Many mothers were sent to other healthcare centres for diagnostic tests*. *If this facility were available within the GPC centre*, *then mothers would not have to go outside*, *and it would reduce the suffering of mothers*.” Another suggestion from the same mother was, “*It would be great if there was a doctor assigned for treating us*.”

A few mothers recommended adding more information on birth control methods. One mother (aged 29) said, “*Discussions about family planning would have been more effective*. *We have just delivered*, *and which contraceptive method would be more effective should be included in GPC*. *They did not discuss this matter much*.”

Participants also suggested to include delivery facility within the GPC programme. A mother (age, 20 years) suggested that, “*It would be more beneficial for mothers if the delivery services were also included in GPC as they checked all the mothers*, *knew their problems and consulted them for nine months*.”

Suggestions about extending staff were also made by the mothers. They felt the necessity of a gynaecologist in the session. One mother (age 21) suggested, “*GPC service would have been more beneficial with availability of specialist doctor (gynaecologist) for the pregnant mother and if all the services- check-up*, *consulting a doctor and diagnostic take place in one centre*.” Another suggestion in this regard by another mother (age 20 years) was, “*A special doctor only for pregnant mothers’ care should be available in GPC*.”

## Discussion

This qualitative study explored the overall experience of pregnant women participating in GPC sessions during their pregnancy period. The analysis identified several themes that expressed the experience of mothers from each aspect of attending GPC sessions. In particular, receiving all services at the same time, social interaction and sharing, reducing waiting time to receive service, friendly behaviour from service providers and other peers, knowledge about pregnancy related health issues with respective advice, and obtaining family support as a result of attending sessions were reported as the benefits of GPC from the attending mothers.

In individual care, the service provider usually has a limited time per patient, and they are sometimes only able to answer a few of the questions asked by patients or provide only essential information during consultation. At times, the information provided by a physician may not meet the underlying needs or questions of pregnant woman [22]. Consequently, patients were also able to ask relatively few questions due to limited visiting times. Sometimes, women might feel shy or uncomfortable asking questions and did not actively participate with service providers during their check-ups [12]. In addition, many women did not have the opportunity to share/learn from others in an individual visit, as they were strangers. However, such constraints were minimised in GPC services. One of the key findings of this GPC experience among mothers is “getting the answers to questions without asking them” (mentioned under theme three). Though this finding does not indicate that all the mothers received answers regarding their questions, the overall findings suggest that women expressed a preference for GPC because it enabled them to learn different health information regarding pregnancy than individual care. In other studies, the central meaning of the GPC experience emerged as “getting more than they realised they needed” [22] or “I was not alone” [23].

Sharing knowledge between peers is another important finding of this study. During sessions, experienced mothers with a previous history of delivery shared their experiences, while first-time mothers benefitted from hearing from them and learned about common discomforts and problems. Another related study also found the same experience from participants [16]. Some of our other findings are supported by previous studies describing the GPC experiences of women, such as reductions in feelings of isolation by sitting together [12,22].

In the present study, an impressive outcome of GPC is that the programme helped to reduce family barriers regarding seeking regular pregnancy care, as described by some participants. An earlier study demonstrated that antenatal care seeking was strongly determined by the knowledge of the mothers-in-law and husbands of women, which were usually the decision makers for ANC [24]. Previously, women in the group faced difficulties in convincing other family members to come for check-ups, though the participants were able to make them understand the importance of maternal healthcare after attending the sessions, later reporting that their family member no more prevented them from going for check-ups after realising their importance. This situation is similar to previous studies that described knowledge helping women to be more conscious about their own health and empowering them to control their healthcare decisions [25–27]. In GPC sessions, many mothers met at one place and moderators discussed different topics in the scheduled discussion. Thus, participants received all of this information and services by sitting there, which was not done in a regular check-up. Participants described this as an opportunity for them and showed positive impressions by delivering pregnancy care in this manner. This type of pregnancy management was also reported as impressive by participants in another study [22]. Group discussions were very beneficial for most mothers since it created a platform for them to learn about their queries—even for those who remained silent or did not ask questions during sessions. Additionally, various questions being asked by women enabled mothers to learn about other topics that might be important to them, but that they forgot to ask or felt too shy to ask. This benefit of GPC was also captured in other studies [12,23]. Care through GPC also strengthened the provider-patient relationship, and this relationship between health care providers and the women in the group was found to be an effective mode of knowledge sharing. Group care reduced the communication gap between women and health care providers while creating a more balanced relationship. By joining the group, women had the opportunity to ask questions in a context where the provider could be clearer. In previous studies, active participation was also observed through group discussion [22,28,29]. Group participants described that they were prepared for labour and delivery, which is consistent with other studies of GPC that showed more knowledge and preparedness related to pregnancy [9,16]. Consistent with our study, higher satisfaction with prenatal care among GPC participants was also found by earlier studies [9,30].

Although participants reported that they received more than what they expected from GPC, some of them expressed the desire for more postpartum and parenting information and care, which is similar to the results of another study [31]. Discussions on family planning, assigning a gynaecologist, and including a delivery service in GPC were the main concerns of the participants. These participant requirements indicate a gap of services that could be addressed to support women in their postpartum and parenting stages.

## Conclusion

Our study explored that pregnancy care in a group is well accepted and preferred by participating mothers in the context of Bangladesh. This approach provides an opportunity for pregnant women to become connected, to share knowledge and experiences, to reduce social isolation, and to learn about pregnancy-related issues in discussion sessions within a supportive environment. However, care should be tailored according to individual needs, whether it occurs in a group or through individual care. The GPC model promises movement towards peer- and family-supported care. The refinement and evaluation of this model should continue to improve the health outcomes of Bangladeshi women. Providers can use the findings of the present study to inform policy makers about the role of the GPC model in improving awareness as well as its impacts on improving female health and service utilisation efficiency. Finally, by addressing the maternal health goal, this study contributes to strengthening service delivery in our existing health care system.

## References

[1] Hogan MC, Foreman KJ, Naghavi M, Ahn SY, Wang M, Makela SM, et al Maternal mortality for 181 countries, 1980–2008: a systematic analysis of progress towards Millennium Development Goal 5. Lancet. 2010;375: 1609–1623. 10.1016/S0140-6736(10)60518-1 20382417

[2] Pulok MH, Sabah MNU, Uddin J, Enemark U. Progress in the utilization of antenatal and delivery care services in Bangladesh: Where does the equity gap lie? BMC Pregnancy Childbirth. 2016;16 10.1186/s12884-016-0970-4 27473150 PMC4967314

[3] National Institute of Population Research and Training (NIPORT) International Centre for Diarrhoeal Disease Research Bangladesh (icddrb) and MEASURE Evaluation. Bangladesh Maternal Mortality and Health Care Survey 2016: Preliminary Report. Dhaka, Bangladesh, and Chapel Hill, NC, USA; 2017.

[4] Lawn JE, Cousens S, Zupan J. Neonatal Survival 1 4 million neonatal deaths: When? Where? Why? 2005; 891–900. 10.1016/S0140-6736(05)71048-5 15752534

[5] National Institute of Population Research and Training (NIPORT). Mitra and Associates and ICF International. Bangladesh Demographic and Health Survey 2014. NIPORT, Mitra and Associates, and ICF International. Dhaka, Bangladesh, and Rockville, Maryland, USA; 2016.

[6] Chowdhury S, Banu L, Chowdhury TA, Rubayet S, Khatoon S. Achieving Millennium Development Goals 4 and 5 in Bangladesh. An Int J Obstet Gynaecol. 2011;118 Suppl: 36–46. 10.1111/j.1471-0528.2011.03111.x 21951501

[7] Sultana M, Mahumud RA, Ali N, Ahmed S, Islam Z, Khan JAM, et al The effectiveness of introducing Group Prenatal Care (GPC) in selected health facilities in a district of Bangladesh: study protocol. BMC Pregnancy Childbirth. 2017;17: 48 10.1186/s12884-017-1227-6 28143611 PMC5282623

[8] Alexander GR, Kotelchuck M. Assessing the Role and Effectiveness of Prenatal Care: History, Challenges, and Directions for Ruture Research. Public Health Rep. 2001;116: 306–16. 10.1016/S0033-3549(04)50052-3 12037259 PMC1497343

[9] Ickovics J, Kershaw T, Westdahl C, Magriples U, Massey Z, Reynolds H, et al Group prenatal care and perinatal outcomes: A randomized controlled trial. Obstet Gynecol. 2007;110: 330–339. 10.1097/01.AOG.0000275284.24298.23 17666608 PMC2276878

[10] Grady MA, Bloom KC. Pregnancy Outcomes of Adolescents enrolled in a Centering Pregnancy Program. J Midwifery Women’s Heal. 2004;49: 412–420. 10.1111/j.1542-2011.2004.tb04435.x 15351331

[11] Novick G. Women’s experience of prenatal care: an integrative review. J Midwifery Womens Health. 2009;54: 226–37. 10.1016/j.jmwh.2009.02.003 19410215 PMC2754192

[12] Novick G, Sadler LS, Kennedy HP, Cohen SS, Groce NE, Knafl KA. Women’s Experience of Group Prenatal Care. Qual Health Res. 2011;21: 97–116. 10.1177/1049732310378655 20693516 PMC3085399

[13] Rising SS. Centering Pregnancy: An Interdisciplinary Model of Empowerment. J Nurse Midwifery. 1998;43: 46–54. PII S0091-2182(97)00117-1 9489291 10.1016/s0091-2182(97)00117-1

[14] Baldwin KA. Comparison of Selected Outcomes of CenteringPregnancy Versus Traditional Prenatal Care. J Midwifery Womens Health. 2006;51: 266–272. 10.1016/j.jmwh.2005.11.011 16814221

[15] Ickovics JR, Kershaw TS, Westdahl C, Magriples U, Massey Z, Reynolds H, et al Group prenatal care and perinatal outcomes: a randomized controlled trial. Obs Gynecol. 2007;110: 330–339. 10.1097/01.aog.0000275284.24298.23 17666608 PMC2276878

[16] Klima C, Norr K, Vonderheid S, Handler A. Introduction of CenteringPregnancy in a Public Health Clinic. J Midwifery Women’s Heal. 2009;54: 27–34. 10.1016/j.jmwh.2008.05.008 19114236

[17] Gorman JR, Usita PM, Madlensky L, Pierce JP. A qualitative investigation of breast cancer survivors’ experiences with breastfeeding. J Cancer Surviv. 2009;3: 181–191. 10.1007/s11764-009-0089-y 19462249 PMC2714446

[18] Colorafi KJ, Evans B. Qualitative Descriptive Methods in Health Science Research. Heal Environ Res Des J. 2016;9: 16–25. 10.1177/1937586715614171 26791375 PMC7586301

[19] Ulin PR, Robinson ET, Tolley EE. Qualitative Methods in Public Health: A Field Guide for Applied Research. San Francisco, USA; 2005. 10.1249/01.mss.0000172593.20181.14

[20] Ganle KK, Parker M, Fitzpatrick R, Otupiri E. A qualitative study of health system barriers to accessibility and utilization of maternal and newborn healthcare services in Ghana after user-fee abolition. BMC Pregnancy Childbirth. 2014;14: 1–17. 10.1186/1471-2393-14-1 25527872 PMC4307897

[21] Braun V, Clarke V. Using thematic analysis in psychology. Qual Res Psychol. 2006;3: 77–101. 10.1191/1478088706qp063oa

[22] McNeil DA, Vekved M, Dolan SM, Siever J, Horn S, Tough SC. Getting more than they realized they needed: a qualitative study of women’s experience of group prenatal care. BMC Pregnancy Childbirth. 2012;12: 17 10.1186/1471-2393-12-17 22436393 PMC3364900

[23] Kennedy HP, Farrell T, Paden R, Hill S, Jolivet R, Willetts J, et al “I Wasn’t Alone”-A Study of Group Prenatal Care in the Military. J Midwifery Women’s Heal. 2009;54: 176–183. 10.1016/j.jmwh.2008.11.004 19410209

[24] Syed U, Khadka N, Khan A, Wall S. Care-seeking practices in South Asia: Using formative research to design program interventions to save newborn lives. J Perinatol. 2008;28: S9–S13. 10.1038/jp.2008.165 19057572

[25] Baldwin K, Phillips G. Voices Along the Journey: Midwives’ Perceptions of Implementing the CenteringPregnancy Model of Prenatal Care. J Perinat Educ. 2011;20: 210–217. 10.1891/1058-1243.20.4.210 22942623 PMC3210635

[26] Ahmed S, Creanga AA, Gillespie DG, Tsui AO. Economic Status, Education and Empowerment: Implications for Maternal Health Service Utilization in Developing Countries. PLoS One. 2010;5: e11190 10.1371/journal.pone.0011190 20585646 PMC2890410

[27] Mahara G, Asweto C, Cao K, Alzain AM, Sebastian A. Utilization of ANC and PNC Services in Nepal: A Multivariate Analysis Based on Nepal Demographic Health Survey 2001 and 2006. Am J Heal Res. 2015;3: 318–327. 10.11648/j.ajhr.20150306.11

[28] Massey Z, Rising SS, Ickovics J. CenteringPregnancy group prenatal care: Promoting relationship-centered care. J Obstet Gynecol Neonatal Nurs. 2006;35: 286–294. 10.1111/j.1552-6909.2006.00040.x 16620257

[29] McDonald SD, Sword W, Eryuzlu LE, Biringer AB. A qualitative descriptive study of the group prenatal care experience: perceptions of women with low-risk pregnancies and their midwives. BMC Pregnancy Childbirth. 2014;14: 334 10.1186/1471-2393-14-334 25258167 PMC4262064

[30] Handler A, Rosenberg D, Raube K, Lyons S. Satisfaction and use of prenatal care: Their relationship among African-American women in a large managed care organization. Birth. 2003;30: 23–30. 10.1046/j.1523-536X.2003.00213.x 12581036

[31] Teate A, Leap N, Rising SS, Homer CSE. Women’s experiences of group antenatal care in Australia-the Centering Pregnancy Pilot Study. Midwifery. 2011;27: 138–145. 10.1016/j.midw.2009.03.001 19386402

