# Correction: Group prenatal care experiences among pregnant women in a Bangladeshi community

**DOI:** 10.1371/journal.pone.0220816

**Published:** 2019-07-31

**Authors:** Marufa Sultana, Nausad Ali, Raisul Akram, Tania Jahir, Rashidul Alam Mahumud, Abdur Razzaque Sarker, Ziaul Islam

The affiliation for the seventh author is incorrect. Ziaul Islam is not affiliated with #5 but with #3: Health System and Population Studies Division, International Centre for Diarrheal Disease Research, Bangladesh (icddr,b), Dhaka, Bangladesh.
